# Trends in child and adolescent obesity prevalence according to socioeconomic position: protocol for a systematic review

**DOI:** 10.1186/2046-4053-3-52

**Published:** 2014-05-26

**Authors:** Alexandra Chung, Kathryn Backholer, Evelyn Wong, Claire Palermo, Catherine Keating, Anna Peeters

**Affiliations:** 1 Obesity and Population Health, Baker IDI Heart and Diabetes Institute, Level 4, 99 Commercial Road, Melbourne VIC 3004, Australia; 2 Department of Epidemiology and Preventive Medicine, Monash University, Level 6, 99 Commercial Road, Melbourne VIC 3004, Australia; 3 Department of Nutrition and Dietetics, Monash University, Level 1, 264 Ferntree Gully Road, Notting Hill VIC 3168, Australia

**Keywords:** Trends, Socioeconomic position, Childhood, Adolescence, Obesity

## Abstract

**Background:**

Obesity is a significant public health issue and is socially patterned, with greater prevalence of obesity observed in the most socioeconomically disadvantaged groups. Recent evidence suggests that the prevalence of childhood obesity is levelling off in some countries. However, this may not be the case across all socioeconomic strata. The aim of this review is to examine whether trends in child and adolescent obesity prevalence since 1990 differ according to socioeconomic position in developed countries.

**Methods:**

An electronic search will be conducted via Ovid Medline, Embase, Cumulative Index to Nursing and Allied Health Literature, Scopus and Cochrane Collaboration to identify articles that report trends in obesity prevalence in children and adolescents according to socioeconomic position. We will also search grey literature databases including the Virtual Library for Public Health and the System for Information on Grey Literature, as well as websites from relevant organisations. Articles that report on a series of cross sectional studies; describe one or more measure of obesity with data recorded at two or more time points since 1990; and report trends by at least one indicator of socioeconomic position will be included. Quality of included studies will be evaluated according to criteria that consider both internal and external validity. Descriptive analysis will be performed to examine trends since 1990 in childhood obesity prevalence according to socioeconomic position.

**Discussion:**

The review will provide a picture of change over time in developed countries of childhood obesity prevalence across socioeconomic strata and identify whether changes in childhood obesity prevalence are experienced equally across socioeconomic groups.

**Systematic review registration:**

PROSPERO CRD42014007625.

## Background

It is well recognised that childhood obesity is a significant public health issue, with adverse physical and psychological effects that persist beyond childhood into the adult years [1]. After decades of rapid increase [2], it appears that childhood obesity prevalence in developed countries is starting to plateau. Reviews of international evidence have shown that the prevalence of obesity in children and adolescents is stabilising in countries including Australia, Japan, France, the UK and US [3,4]. However, evidence also suggests that such progress may not have been shared among children across all socioeconomic groups [4,5].

An international systematic review published in 2010 [4] examined obesity prevalence trends and reported levelling off of the obesity epidemic in recent years. Heterogeneity in obesity trends were reported across socioeconomic strata, with levelling of obesity prevalence less apparent for more disadvantaged socioeconomic groups [4]. However, the authors noted that trends by socioeconomic strata were only explored in a small number of their included studies [4]. Individual studies reporting the impact of socioeconomic position (SEP) on obesity prevalence provided mixed results. Studies from Australia [5] and England [6] reported socioeconomic differences in obesity trends among children and adolescents, while evidence from France [7,8] did not show a difference. With a specific focus on SEP and childhood obesity, this review will capture additional data, including papers published since 2010, to allow greater understanding of trends in the prevalence of obesity by SEP.

Further investigation is warranted, particularly because of the existing excess burden of obesity in children in a lower SEP. Given the health risks associated with excess weight, and the observed socioeconomic patterning in chronic diseases, if trends in obesity prevalence are not improving at the same rate across socioeconomic groups, this will likely lead to further inequalities across a range of health and wellbeing outcomes. Understanding the differences between subgroups of the population is critical to ensuring policy makers can make informed decisions as to where preventive efforts should be focused. This is particularly important in light of evidence that demonstrates differential effectiveness of a number of obesity prevention interventions according to SEP [9].

The aim of this review is to examine whether trends in child and adolescent obesity prevalence since 1990 differ according to socioeconomic position in developed countries.

## Methods

### Literature search strategy

The search strategy will include searches of the following electronic databases: Ovid Medline, Embase, Cumulative Index to Nursing and Allied Health Literature, Scopus and Cochrane Collaboration. Databases will be searched for articles published between January 1990 and February 2014. We will also search grey literature databases including the Virtual Library for Public Health and the System for Information on Grey Literature, as well as websites from relevant organisations. Finally, we will hand-search reference lists of all included articles. As a proxy for developed countries, we will focus the search on literature from countries that are members of the Organisation for Economic Co-operation and Development (OECD).

### Search terms

Search terms will include relevant medical subject headings (MeSH) and keywords in the title, abstract and text for terms including overweight, obesity, socioeconomic position, children and OECD member countries (see Table 1). The search will be limited to studies published in English since 1990.

**Table 1:** Search terms

**Concept**	**Search terms**
Overweight, obesity	*MeSH terms*: overweight/obesity/body mass index/*Free-text terms*: overweight, obesity, body mass index, BMI, body weight, waist circumference, waist hip ratio, adiposity, anthropometric
Socioeconomic position	*MeSH term*: Socioeconomic factors/ *Free-text terms:* socioeconomic factors, socioeconomic, socio-economic, socioeconomic status, socioeconomic gradient, social class, social gradient, social inequalit*, inequalit*, disparit*, disadvantage*, poverty, income, employment status, education* status, educational attainment, deprivation, health inequalit*
Childhood	*MeSH terms:* child/child, preschool/adolescent/ *Free text terms*: child*, adolesc*, school
OECD countries	*MeSH term*: developed countries/*MeSH exp & Free-text* for each OECD member country (America, Australia, Austria, Belgium, Canada, Chile, Czech Republic, Denmark, England, Estonia, Finland, France, Germany, Great Britain, Greece, Hungary, Iceland, Ireland, Israel, Italy, Japan, Korea, Luxembourg, Mexico, Netherlands, new Zealand, Norway, Poland, Portugal, Slovak Republic, Slovakia, Slovenia, Spain, Sweden, Switzerland, Turkey, United Kingdom, United States)
OECD, Organisation for Economic Co-operation and Development.	

### Inclusion criteria

Articles will be included if they report socioeconomic trends in the prevalence of obesity in children and/or adolescents aged 2 to 18 years from at least two time points since 1990. Socioeconomic markers could include one or more family- (parent education, parent occupation, family income) or area-level (household postcode, school or neighbourhood socioeconomic index) indicator. Obesity markers will include at least one measured or self reported anthropometric measure (weight and height, body mass index (BMI), BMI z-scores, height and weight plotted on growth charts/percentile charts, waist circumference, waist to hip ratio, percentage body fat, skinfold thickness). Only studies from OECD member countries (chosen as a proxy for developed countries) will be included.

### Exclusion criteria

Cohort studies that report time trends not independent of aging will be excluded. Clinical studies, obesity intervention or treatment studies and studies conducted among single or high-risk groups such as low socioeconomic populations or ethnic minorities will be excluded.

### Study selection

The initial screening of titles and abstracts will be completed independently by two authors. Full text articles will then be retrieved and assessment against inclusion criteria and data extraction will be conducted independently by two authors, using an electronic spreadsheet. Any discrepancies will be resolved through discussion with a third author.

### Data extraction

From the included publications we will extract, where available: author; journal; year of publication; location of study (country/state/city); survey years and time points of data collection; sample population (national survey/community survey/school); sample size; response rate or participation rate; age of population; measure of overweight and/or obesity and whether this is measured or self reported; indicator of SEP and whether this is a family- or area-level marker; any other stratification of results; descriptive results including time trends and obesity prevalence according to SEP; results of significance testing for differences in trends.

### Quality assessment

The quality of included studies will be evaluated independently by two authors, according to criteria adapted from an existing quality assessment tool for quantitative studies from the Effective Public Health Practice Project [10]. We will descriptively assess internal and external validity of included studies with questions on selection bias, study design, confounders, data collection and data analysis (see Table 2). We will perform a sensitivity analysis to evaluate the potential effect of study quality on our conclusions by repeating our analysis on only those studies with high quality ratings for all components.

**Table 2:** Quality assessment

**Component**	**Questions**	**Assessment**	**Ratings**
Selection bias	Are the individuals selected to participate likely to be representative of the national population?	• Very likely	Studies that are very likely to be representative and have greater than 80% participation will be rated as strong.
		• Somewhat likely	
		• Not likely	
		• Cannot tell	
	What percentage of selected individuals agreed to participate?	• 80 to 100%	
		• 60 to 79%	
		• Less than 60%	
		• Can’t tell	
Study design	Were study methods comparable over time?	• Yes	Studies with comparable methods over time will be rated as strong.
		• No	
		• Cannot tell	
Confounders	Were confounders (age, sex, race/ethnicity) controlled for in study design or analysis?	• Yes	Studies that control for confounding will be rated as strong.
		• No	
		• Cannot tell	
Data collection methods	Was anthropometry measured (as opposed to self reported)?	• Yes	Studies where anthropometry was measured will be rated as strong.
		• No	
		• Cannot tell	
Analyses	Are the statistical analyses appropriate to detect differences by SEP?	• Yes	Studies that have performed analyses to detect differences by SEP will be rated as strong.
		• No	
		• Cannot tell	
SEP, socioeconomic position.			

### Data synthesis

We will report on trends in child and adolescent obesity prevalence according to SEP and discuss whether trends are homogenous across the socioeconomic strata. Where trends have been reported by more than one marker of SEP we will preferentially select the marker of SEP that is found to be most common among included articles for our primary analysis. Secondary analysis will consider other reported SEP measures to examine any differences in findings according to SEP measure used. Secondary analysis will also be undertaken to explore any additional available obesity-related outcome data (for example, BMI) to examine the continuous relationship between SEP and excess weight.

We will generate summary tables, firstly using crude data on prevalence from all articles and then, where data are available, we will table results of studies that have undertaken significance testing of differences in trends. Analyses will be conducted to examine overall trends as well as trends over specific time periods. We will also analyse variations in findings according to country, age group (childhood, adolescence) and sex, and will discuss trends in terms of both absolute and relative inequalities where possible.

## Discussion

In this review we will examine studies of child and adolescent obesity prevalence published since 1990 in order to analyse and compare trends across different socioeconomic strata. The findings will provide a comprehensive picture of recent trends in child and adolescent obesity prevalence in developed countries according to SEP, contributing to a greater understanding of the relationship between SEP and childhood obesity. Further, the review will provide evidence to help understand any socioeconomic disparities in childhood obesity trends and reveal if current reporting of the recent plateau in obesity prevalence masks important differences across the socioeconomic strata. In so doing the findings of this review will contribute to evidence-based policy making including policy decisions to reduce obesity-related inequalities.

## Abbreviations

BMI: body mass index; OECD: Organisation for Economic Co-operation and Development; SEP: socioeconomic position.

## Competing interests

The authors declare that they have no competing interests.

## Authors’ contributions

AC shared responsibility for the development of the research question and the research approach and drafted the article. KB, EW, CP and CK contributed to the development of the protocol and manuscript. AP shared responsibility for the development of the research question and the research approach, and contributed to the development of the protocol and manuscript. All authors read and approved the final manuscript.
